# Creating New Forms of Hexaploid Triticale Associating Complete R and D Genomes

**DOI:** 10.3390/biology14111632

**Published:** 2025-11-20

**Authors:** Michel Bernard, Sylvie Bernard, Ekaterina Badaeva, Rolf Schlegel

**Affiliations:** 1 Department of Genetics, Diversity and Ecophysiology of Cereals, Institut National pour la Recherche Agronomique et l’Environnement (INRAE), 63000 Clermont-Ferrand, France; 2 Vavilov Institute of General Genetics, Russian Academy of Sciences, Moscow 119991, Russia; katerinabadaeva@gmail.com; 3 Department of Resistance Research and Stress Tolerance, Julius Kühn Institute, 06484 Quedlindburg, Germany; rolf.schlegel@t-online.de

**Keywords:** triticale, introgression, evolution, polyploidy, D genome, wheat, rye, chromosomes, FISH, *Aegilops tauschii*

## Abstract

Triticale, a man-made cereal, has been grown worldwide since the 1980s in order to replace established cereals in difficult areas, at least partially. The present cultivars are mostly hexaploid genotypes with 42 chromosomes, of genomic structure AA BB RR. Their agricultural performance does not meet all breeding requirements. In particular, some technological characteristics are inadequate compared to tetraploid (durum) and hexaploid (soft) wheats. Therefore, we aimed to find ways to improve modern triticale varieties by targeted introgression with genes and even chromosomes from wheat, in particular, from the D genome. Through appropriate bridge crossings and embryo culture technique and under cytogenetic control, a series of new stable hexaploid lines with reasonable agronomic stability were finally produced. All of them carried a complete D sub-genome, a complete R sub-genome, plus a mixed genome consisting of various combinations of chromosomes derived from the A and B genomes representing the seven homoeologous groups. It is clear that such mixed genomes can be of genetic and breeding significance. These large introgression lines demonstrate the flexibility of genome organization and offer the opportunity for further regulatory and genetic optimization.

## Simple Summary

The aim of this work was to create a new type of hexaploid Triticale (2*n* = 6*x* = 42), possessing the complete D sub-genome of wheat (2*n* = 6*x* = 42, BB AA DD) originating from *Aegilops tauschii*, the complete sub-genome of rye (2*n* = 2*x* = 14, RR), and a mixogenome consisting of seven chromosome pairs derived from the A and B sub-genomes of wheat, in order to introduce new technological properties controlled by D genome, and genes for disease resistance into triticale.

## 1. Introduction

Triticale is a type of cereal crop that was added in the 1980s to the established grain crops such as wheat, rye, or barley. Triticale breeding has been developed for more than a hundred years since the wheat and rye were first merged around 1875. This was difficult because hybridization between distantly related species, such as wheat and rye, leads to almost sterile products, since the chromosomes of the two species cannot pair with each other during meiosis. Incomplete fertility of the plants is achieved by colchicine doubling the A, B, D, and R genomes. In addition, it turned out that hexaploid triticale (AABBRR) are more agronomically suitable than the octoploid forms (AABBDDRR) [1], although the quality of their flour was unsatisfactory compared to tetraploid (durum, AABB) and hexaploid (soft, AABBDD) wheat parents. This is caused by the lack of important genes associated with baking quality and located in the D sub-genome of hexaploid wheat. Therefore, triticale varieties are mainly used as a forage crop, as triticale is adapted to poor soils and its grains have, on average, slightly higher protein content compared to wheat.

**Figure 1:**
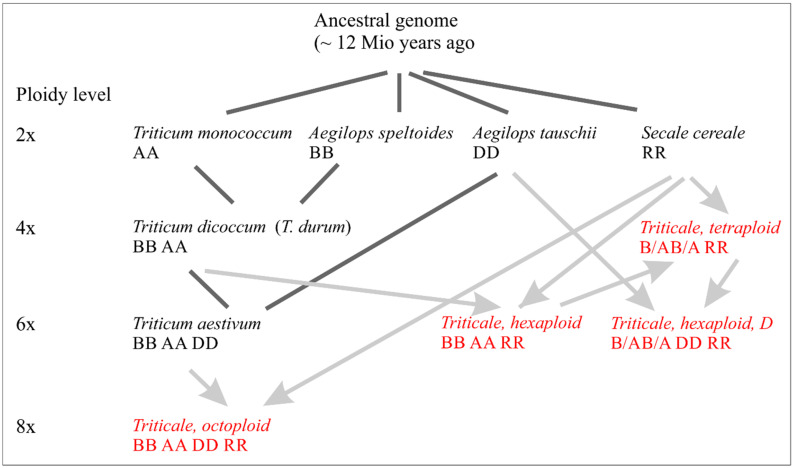
Simplified scheme of origin of some wheat and rye species (black), including artificial stabilized hybrids between them (red).

The aim of our research was to adjust the genomic structure of triticale in such a way that, in addition to high fertility, i.e., yield, a better nutritional quality of the product will be achieved. This means that the genome structure that has been formed and optimized over millions of years must be modified [2,3,4,5,6]. According to the current concept of phylogeny, a large number of diploid and polyploid species arose from a postulated primordial genome (Figure 1) [7].

The polyploid wheat species carry at least the A and B sub-genomes. The A sub-genome derives from the diploid wild einkorn wheat *Triticum urartu* [8]. The B genome comes from the wild grass *Aegilops speltoides* (the genome formula considers the cytoplasm of *Aegilops speltoides* as host of the wheat genomes) [9]. The fusion of the A and B genomes, approximately 0.800–0.500 MY ago, gave rise to wild tetraploid species *Triticum dicoccoides,* from which the tetraploid cultivated forms, i.e., emmer *T. dicoccum*, durum wheat (*T. dicoccum* ssp. *durum*, AABB), and others were derived. The other tetraploid species, *Triticum timopheevi* (GGA^t^A^t^), occurred independently from the same parental species nearly 0.300 MY ago.

Rye (*Secale cereale*), which contributed the R genome into triticale, is a diploid species. It evolved from the supposed common ancestor of the Triticeae through the accumulation of mutations and specific DNA sequences (especially transposons) as well as through chromosomal rearrangements [10,11]. Compared to the wheat parents, rye is characterized by the allogamous pollination mode (self-sterility), greater cold tolerance, better resistance to various diseases, low soil requirements, and other useful properties. They should be combined with those of wheat in a hybrid form artificially created by the breeder, i.e., triticale [12] (Figure 1).

The first commercially successful Triticale varieties were “Clercal” (France) and “Lasko” (Poland), released in the 1980s. Clercal was selected from crosses between primary octoploid forms and hexaploid ‘varieties’ from Hungary and Canada obtained since the 1960s, as well as subsequent drastic selection with regard to increased spike fertility, good grain filling, and improved lodging tolerance of the stalks. Since the 1990s, the acreage of rye in France has declined (2021: 31,000 ha), while the acreage of triticale has subsequently increased to 330,000 ha. In other countries where rye was the main cereal, triticale extension could be even greater (Poland).

Modern triticale breeding is focused on the improvement of grain quality by incorporating the D sub-genome; this genome originally derived from *Ae. tauschii* (syn. *Ae. squarrosa*), but adapted to polyploidy forms and cultivation. It also offers opportunities for optimization of other traits such as early maturity, photoperiod insensitivity, technological properties, and resistance to rust diseases or stem reduction of triticale. This was to be attempted in different ways.

An approach could be the induction of genetic recombination between the R and D genome chromosomes; however, naturally homoeologous chromosome pairing is almost impossible [13,14]. This handicap could be circumvented by means of suitable bridge crossings and promotion of spontaneous D/R chromosome substitutions. Substituting individual rye chromosomes with chromosomes from the D sub-genome is interesting—from a breeding perspective—but not easy to achieve. For example, CIMMYT breeders (Mexico) were able to obtain hexaploid Triticale cv. “Armadillo”, which carries a spontaneous 2D (2R) chromosome substitution (Rajaram, pers comm).

However, the existence of tetraploid triticale derived from crosses between hexaploid triticale and rye, and associating the R genome with a “mixogenome” (x = 7) combining A and B chromosomes (A/B, symbol: Љ), offers a new perspective [15,16]. In fact, it appears possible to directly combine this form with *Ae. tauschii,* the diploid D genome donor, in order to produce new hexaploid genotypes.

## 2. Materials and Methods

### 2.1. Creating Primary Amphiploids DT4x

**Figure 2:**
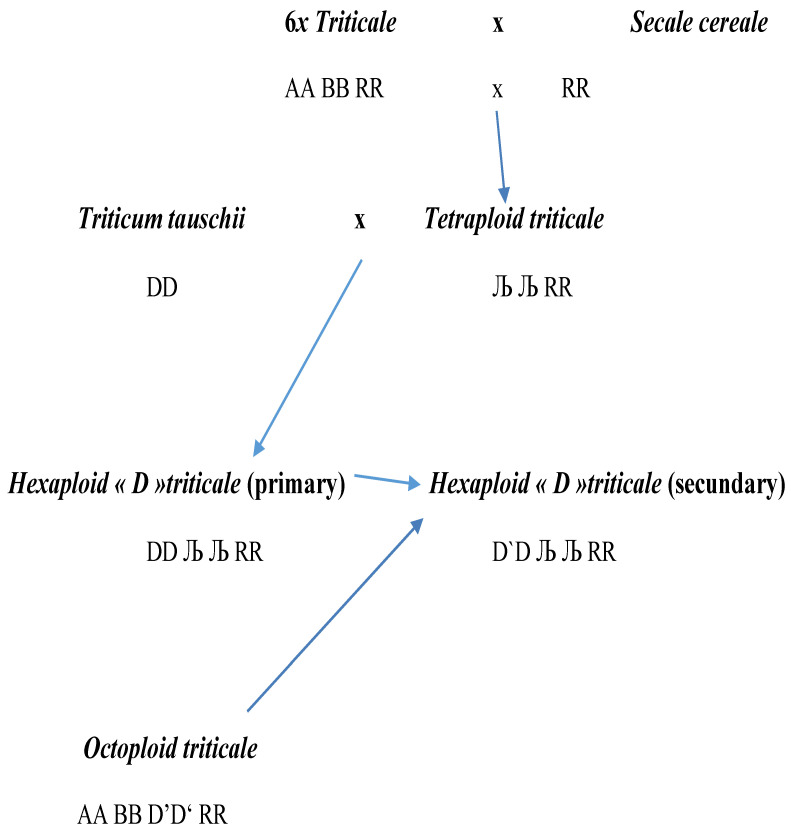
Crossing scheme for development of recombined hexaploid triticales including A, B, D, and R genome chromosome.

To add the D genome to the existing tetraploid triticale, seven lines of *Ae. tauschii* were used, kindly provided by B.S. Gill (Wheat Genetic Resource Center, Kansas State University, Manhattan, KS, USA): Ku2080, Al8-78, Ku1574-72, Ku2131, Ku2101, Ku2115, Ku2124. They served as maternal parents in crosses with a viable, fertile, and already reshaped tetraploid triticale (ЉЉ RR; Figure 2). We used an F5 T4*x* line obtained by backcrossing a primary tetraploid triticale (T4*x*) with a good hexaploid triticale cultivar (AABBRR, Clercal sister line). This T4*x* line had the chromosome constitution (1A 2A 3B 4B 5B 6B 7A and 1R 2R 3R 4R 5R 6R 7R) at the diploid level, which was verified by Giemsa C-banding and a series of microsatellite markers [17,18]. Hybrid embryo rescue followed by chromosome doubling by colchicine was carried out according to the methodology of Bernard and Bernard [19].

### 2.2. Creating Secondary Forms Introgressing the D Genome of Wheat

These hexaploid offspring (DD ЉЉ RR) were backcrossed with four stable breeding lines of octoploid triticale (2*n* = 8*x* = 56, AABBD`D`RR) from the Station Agronomique de Changins (Nyon, Switzerland): MtCalm3, MtCalm7, MtCalm47, and MtCalm82 (Figure 2), resulting in heptaploid hybrids (A Љ B DD’ RR). In their progeny, several lines with 42 chromosomes have been isolated, and some of them were characterized by fluorescence in situ hybridization (FISH) [20,21].

## 3. Results

With the help of embryo rescue, 18 F1 hybrid plants were obtained from crosses between *Ae. tauschii* (2*n* = 2*x* = 14, DD) and the tetraploid (2*n* = 4*x* = 28, ЉЉ RR) triticale line. The reciprocal crosses proved to be less favorable. The chromosomes of the F1 plants (2*n* = 21, Љ D R) were doubled through colchicine treatment in order to restore spike fertility. In this way, forty offspring were finally available in 2006–2007, each with 2*n* = 6*x* = 42 chromosomes (DD ЉЉ RR). Morphologically, they appeared rather weak, including low fertility. The glumes were very hard and tough. Only six of these amphidiploids were sufficiently vigorous so that enough offspring could be produced. Three of them were then crossed with octoploid triticale.

From ten crosses combining these 6*x* and 8*x* amphidiploids, 20 F1 seeds were obtained that had 2*n* = 7*x* = 49 chromosomes (heptaploid genome: A B Љ DD’RR). They were reasonably fertile and produced 70 F2 seeds, of which only 50 grew up to adult plants (Figure 2).

**Figure 3:**
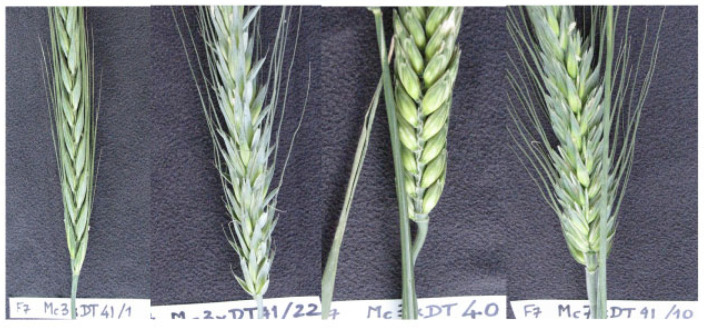
Some spike samples of segregating F8 triticales from crosses of diploid *Aegilops tauschii* (DD) × recombined tetraploid triticale (ЉЉ RR) and backcrossing to octoploid triticale (BBAAD’D’RR). (Lines: Mc3xDT41/1; Mc3xDT41/22; Mc3x DT40; Mc7xDT41/10).

From these 50 plants, 39 were fertile and produced an F3 offspring. Their progenies could be developed up to the F8 generation. All of them were subjected to strict selection for fertility, spike morphology, and grain quality. Thus, twenty F8 offspring were derived from three original crosses of DT4xxT8x (Mc3xDT41, Mc7xDT41, Mc5xDT40), leading to new breeding lines (Figure 3).

**Figure 4:**
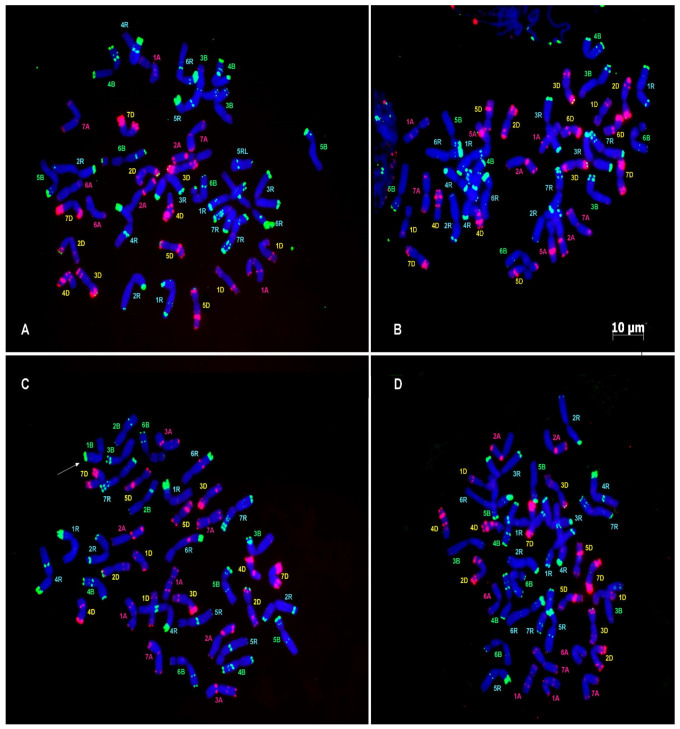
Metaphase cells of hexaploid triticale forms (**A**) F_7_Mc3xDT41/2-1; (**B**) F_7_Mc3xDT41/10; (**C**) F_7_Mc7xDT40-3; (**D**) F_7_Mc5xDT40-3b. Chromosomes were labeled with probe combinations oligo-pSc119.2 (green) and oligo-pTa535 (red). Chromosomes were classified according to genetic nomenclature and designated with pink (A-genome), green (B-genome), yellow (D-genome), and turquoise (R-genome) letters. Arrow point on extra-1B (**C**). Scale bar, 10 µm.

Cytogenetic analysis of the F8 lines showed that they are true secondary triticales with the genomic formula ЉЉ DD’RR. All contain a diploid D genome, which must have arisen from the recombination between homologous D chromosomes (from *Ae. tauschii*, and from the octoploid triticale (AA BB D’D’ RR)) (Figure 4). However, chromosomes from the A and B genomes could replace individual rye chromosomes (mainly 3R) or D chromosomes (Table 1).

There are some cytological differences between lines. One line (Mc5xDT40-3b) had a different chromosome 7R; the chromosome 5R of (Mc7xDT40-3) lost the telomeric pSc119.2 cluster (heterochromatin) in the short arm, and the chromosome 6R does not look normal (it has no telomeric pSc119.2 cluster in the short arm and a clear pTa535 site in the long arm). The chromosome 3B of (Mc3xDT41-2) and (Mc5xDT40-3b) has very faint pSc119.2 signals in the short arm, but they are rather intense in two other lines. The chromosome 6B of (Mc7xDT40-3) carries a clear pSc119.2 signal in the long arm, but the respective loci were faint in (Mc3xDT41-2) and (Mc5xDT40-3b). Plant (Mc7xDT40-3) carries an extra 1B in addition to a pair of 1A chromosomes. These observations suggest there was some homoeologous recombination and competition between homoeologous chromosomes in the previous generations.

These established secondary triticales already have some advantageous properties that were not found in the raw DT4*x* parental triticale lines (better fertility, easy threshing, soft glumes, semi-compact spikes). They seem phenotypically stable; thus, they offer a good basis for advanced breeding.

This opens up the possibility, for example, that the *Ha* gene, which contributes to the improvement of grain hardness, can be introgressed [22,23]. It is located on the short arm of chromosome 5D and tightly linked or identical to a gene controlling the level of extractable free polar lipids in the grain, Fpl-1. Similarly, the *Glu* genes from the D genome could be introduced in a triticale background, as well as rust resistance genes, or other genes of interest.

## 4. Discussion

As shown in the present work, it is possible to transfer all or a major part of the D genome (either from *Ae. tauschii* or hexaploid wheat) into a synthetic hexaploid triticale via adapted bridge crosses. It is expected that the D genome of *Ae. tauschii* can genetically recombine with the D genome of hexaploid wheat (*Triticum aestivum*, AABBD’D’), adapted to polyploidy. In one case, the “Mc3xDT41-10” family, the D genome was completely integrated, in the other cases (Mc7xDT40-3, Mc5xDT40-3b, Mc3xDT41-2) some chromosomes (particularly 6D) could be substituted by their homoeologous A or B, respectively (Table 1).

The fact that rye chromosomes (2R, 3R particularly) were also substituted in some of the progeny is partly explainable. They seem to be less well tolerated in a wheat genetic background. When obtaining wheat-rye addition lines, the 3R addition is often difficult to maintain [24,25]. In some hexaploid triticale varieties (“Towan,” “Tyalla,” “Caborca 79,” and “Armadillo”), the rye chromosome 2R was spontaneously replaced by the wheat chromosome 2D [26]. This may be due to the differential evolution of the chromosomes between genomes of wheat and rye over the course of 13 million years of evolution.

In all four introgression lines analyzed (Mc7xDT40-3, Mc5xDT40-3b, and Mc3xDT41-2 and 10), some A, B, and R genome chromosomes were also observed in a monosomic condition. This could cause cytological problems, i.e., uneven chromosome distribution during meiosis, leading to various aberrations. On the other hand, they occasionally offer the possibility for homoeologous chromosome substitution, or pairing and recombination. Such events were earlier detected in primary crosses between wheat and rye [1]. They could be optimized [27] by new techniques.

The fact that the tetraploid triticales and these derived hexaploid “DT4x” lines are made of a mixture of A and B genome chromosomes proves that triticale does not necessarily have to consist of complete A, B, D, and R genomes. It has been previously described that the genomes of wheat and rye carry a lot of redundant genetic information [28,29,30]. This redundancy could allow essential genes to be conserved in artificial structures, by organized “bootstrapping” of chromosomes, without modification of the genome’s basic number. The degree of homoeology between the different genomes of wheat and rye, modern or ancestral, ensures this possibility [31]. With advancing genetic technology, the fine-tuning and/or regulation within and between the genomes of the recombinant introgression lines will then be better understood and optimized.

Therefore, it is clear that the D and R genomes can cohabit in a hexaploid structure, together with a third genome that is ‘compound’ (mixogenome). It is interesting to note that the primary raw structures AADDRR or BBDDRR have never been obtained until now. This A/B mixogenome, coded Љ, seems essential, being constituted by two subgroups, assembling some A elements from one part, B elements from the other, or from their symmetric (1, 2, and 7 from the first, 3, 4, 5, and 6 from the other, or reciprocally). This observation suggests that the two subgroups are functional, complementary, and interchangeable units. We can therefore assume that they contribute essential elements of information for the organization of this mixogenome. Obviously, it will be interesting to analyze the specific functioning of this new entity.

Then, the question remains whether it is possible to establish ‘pure’ structures like AADDRR or BBDDRR. This can be performed by crossing symmetrical (complementary) tetraploid triticales. If not, it would imply that the joint presence of particular A and B elements is compulsory. This could open new ways to understand the emergence of high-level amphiploid structures from lower levels, diploid or tetraploid.

## 5. Conclusions

Therefore it was shown that it is possible to associate R and D genomes in an hexaploid structure, and then to dispose of a triticale with better baking properties, and which can be a bridge between wheat and classical triticale.

## Figures and Tables

**Table 1:** Compilation of chromosome constitutions of four F8 progenies of triticale from crosses of diploid *Aegilops tauschii* (DD) × tetraploid triticale (Љ Љ RR) and backcrossing to octoploid triticale (BB AA D’D’RR) revealed by FISH analysis. (a) Line Mc3xDT41-2, (b) Line Mc3xDT41-10, (c) Line Mc7xDT40-3, (d) Line Mc5xDT40-3b (from top to bottom). (+ means chromosome present, - it is absent).

(a) Mc3xDT41-2							
Genome	Chromosome						
	1	2	3	4	5	6	7
A	++	++	--	--	--	++	++
B	--	--	++	++	++	++	--
D	++	++	++	++	++	--	++
R	++	++	++	++	+/5RL	++	++
(b) Mc3xDT41-10							
Genome	Chromosome						
	1	2	3	4	5	6	7
A	++	++	--	--	++	--	++
B	--	--	++	++	++	++	--
D	++	++	++	++	++	++	++
R	++	++	++	++	--	++	++
(c) Mc7xDT40-3							
Genome	Chromosome						
	1	2	3	4	5	6	7
A	++	++	++	--	--	--	++
B	+-	++	++	++	+	++	--
D	++	++	++	++	++	--	++
R	++	++	--	++	++	++	++
(d) Mc5xDT40-3b							
Genome	Chromosome						
	1	2	3	4	5	6	7
A	++	++	--	--	--	++	++
B	--	--	++	++	++	++	--
D	++	++	++	++	++	--	++
R	++	++	++	++	++	++	++

## Data Availability

The created plant material is available at INRAE Clermont Ferrand (France), Genetics, diversity and ecophysiology of cereals.
